# From compliance to innovation: Reframing occupational noise-induced hearing loss prevention in Africa through health equity and the Fourth Industrial Revolution

**DOI:** 10.4102/sajcd.v73i1.1182

**Published:** 2026-09-15

**Authors:** Katijah Khoza-Shangase

**Affiliations:** 1 Department of Audiology, Faculty of Human and Community Development, University of the Witwatersrand, Braamfontein, South Africa

**Keywords:** artificial intelligence, digital health, health equity, hearing conservation, occupational noise-induced hearing loss, tele-audiology, Fourth Industrial Revolution (4IR), sub-Saharan Africa

## Abstract

Occupational noise-induced hearing loss (ONIHL) remains an insufficiently addressed public health concern in sub-Saharan Africa. Despite occupational health and safety regulations and Hearing Conservation programmes (HCPs), prevention remains compliance-driven and unevenly implemented. Global evidence indicates increasing hearing loss, with sub-Saharan Africa facing compounded risks from occupational noise, infectious diseases and socio-economic inequities. This opinion paper advocates innovation-led, equity-driven ONIHL prevention integrating Fourth Industrial Revolution (4IR) technologies within hearing conservation systems. The article critically reflects on current occupational hearing conservation approaches and examines tele-audiology, mobile health (mHealth), artificial intelligence (AI), predictive analytics, Internet of Things (IoT)-enabled monitoring and big data as tools for strengthening prevention. Existing HCPs have strengthened occupational surveillance and regulatory awareness, but gaps remain in predictive risk monitoring, continuity of care, multi-hazard integration and inclusion of informal and underserved populations. Fourth Industrial Revolution technologies could decentralise services, strengthen exposure surveillance and early risk identification and expand access. They should augment, rather than replace, existing HCPs. Implementation must account for inconsistent connectivity, limited device access and variable digital literacy. Hybrid digital, community-based and workplace-supported models may therefore be more feasible. Ethical governance, digital sovereignty, trade union engagement and policy reform are central to equitable implementation.

**Conclusion:**

Repositioning ONIHL prevention within a health equity and innovation framework can strengthen hearing conservation systems while advancing sustainable and socially just hearing health strategies.

## Introduction

Noise-induced hearing loss (NIHL), defined as permanent sensorineural hearing impairment resulting from prolonged exposure to hazardous noise levels, remains one of the most persistent yet preventable occupational and environmental health challenges globally (Anim et al., 2023; Khoza-Shangase, 2025a; World Health Organization [WHO], 2021). Occupational noise-induced hearing loss (ONIHL) specifically refers to hearing damage acquired through workplace-related noise exposure, commonly within mining, manufacturing, transport, construction and other high-risk industrial settings (Moroe, 2020). In sub-Saharan Africa (SSA), ONIHL continues to represent a substantial occupational and public health burden (Anim et al., 2023; Khoza-Shangase, 2025a). Despite decades of occupational health and safety (OHS) regulation and implementation of Hearing Conservation Programmes (HCPs), prevention frameworks remain largely compliance focused, fragmented and insufficiently responsive to African socio-economic realities (Ibili et al., 2023; Moroe, 2020).

Within this article, ‘compliance’ refers primarily to adherence to legislated occupational noise exposure limits, mandatory audiometric surveillance, hearing protection requirements and reporting obligations embedded within national OHS and mining regulations. Monitoring is typically undertaken through employer-led HCPs, occupational health practitioners, labour inspectorates and compensation systems. However, the existence of regulations and HCPs does not necessarily translate into effective prevention outcomes or equitable implementation across sectors and worker populations (Khoza-Shangase, 2025a). These systems often marginalise audiologists, inadequately address workers outside well-resourced formal industries and insufficiently integrate environmental and social determinants of hearing health, resulting in inequities in access to prevention, surveillance and follow-up services (Khoza-Shangase, 2026; Moroe & Khoza-Shangase, 2018; Pillay, 2020; WHO, 2021). Within this article, health equity refers to reducing avoidable and unfair differences in access to hearing conservation and hearing healthcare among populations with differing occupational and socio-economic circumstances (WHO, 2021). This interpretation aligns with broader public health concepts of equity rather than suggesting the absence of occupational legislation.

The urgency of reframing ONIHL prevention is highlighted by recent epidemiological evidence. The Global Burden of Disease (GBD) 2021 study reports a sustained rise in age-related and other hearing loss globally over the past three decades (Ferrari et al., 2024; Zhu et al., 2025). In SSA, this trend coexists with persistent infectious contributors such as otitis media, human immunodeficiency virus (HIV) and tuberculosis, resulting in a compounded risk landscape for auditory impairment (Khoza-Shangase, 2025b; Khoza-Shangase & Sebothoma, 2025). Within this context, ONIHL represents a critical and preventable contributor to long-term disability. Although broader African examples are included where relevant, the primary focus of this opinion paper is SSA, particularly low- and middle-income contexts where occupational exposure, healthcare inequities and resource constraints intersect most acutely.

This opinion paper is directed towards audiologists, occupational health practitioners, policymakers, researchers, industry stakeholders and public health professionals involved in occupational hearing conservation and prevention planning. The article argues that SSA must move beyond compliance towards innovation, embedding health equity at the centre of prevention.

The Fourth Industrial Revolution (4IR) refers to the integration of advanced digital, biological and physical technologies that enable automation, real-time connectivity, intelligent systems and data-driven decision-making across sectors (Schwab, 2016). Within healthcare, 4IR technologies include telehealth platforms, artificial intelligence (AI), Internet of Things (IoT)-enabled devices, predictive analytics, mobile technologies and big data systems that can support decentralised and responsive service delivery. Tele-audiology, mHealth, AI, predictive analytics and IoT-enabled monitoring offer the potential to decentralise hearing conservation, strengthen existing HCPs, extend protection to underserved populations and address structural barriers to implementation (Khoza-Shangase, 2025c, 2025d).

## Africa’s compliance problem: Fragmentation, inequity and structural gaps

The persistence of ONIHL in SSA reflects systemic design limitations rather than resource scarcity alone (Booi, 2020; Khoza-Shangase, 2022, 2025e; Moroe, 2020). Existing occupational health and HCPs have contributed meaningfully to occupational hearing surveillance and regulatory compliance within many formal industries. However, important implementation gaps remain (Khoza-Shangase & Moroe, 2020; Moroe & Khoza-Shangase, 2018), particularly regarding predictive surveillance, continuity of care, multi-hazard monitoring and equitable access across diverse occupational settings (Khoza-Shangase & Moroe, 2022; Moroe & Shandu, 2026).

In South Africa’s mining sector, despite legislated occupational exposure limits of 85 dBA, many workers continue to experience hazardous levels (Chendume, 2021). Meta-analytic evidence demonstrates high prevalence rates of hearing impairment in high-risk mining zones (Anim et al., 2023), with vulnerability compounded by synergistic exposures including whole-body vibration, dust and ototoxic medications (Khoza-Shangase, 2025b; Madahana et al., 2024). Current HCPs often inadequately integrate these multiple occupational and health risks into prevention and monitoring frameworks, limiting opportunities for early intervention and comprehensive risk management (Khoza-Shangase & Sebothoma, 2025). Informal workers (i.e. individuals working outside formally regulated employment arrangements, including many self-employed and small-scale enterprises), who constitute a substantive proportion of the labour force across SSA, remain insufficiently reached by structured occupational HCPs despite existing legislative protections in many jurisdictions (Khoza-Shangase, 2025a, 2025f; WHO, 2021). This reality highlights an important implementation gap within current prevention systems, particularly as many HCPs were originally designed for formal industrial settings and not broader community-linked occupational contexts (Kirsten, 2024).

Beyond auditory effects, chronic occupational noise exposure contributes to cardiovascular stress, metabolic dysregulation, cognitive fatigue and impaired reaction times (Mehrotra et al., 2024). These non-auditory consequences increase accident risk and may negatively affect productivity and workplace safety. However, they are not routinely integrated into conventional occupational hearing conservation monitoring systems (Basner et al., 2014; Mehrotra et al., 2024). Occupational noise-induced hearing loss in SSA therefore reflects not only a clinical concern, but also broader structural inequities in occupational health protection. The unequal distribution of occupational hazards across socio-economic groups further reinforces disparities in hearing health outcomes and access to preventative services.

These observations do not suggest that current HCPs lack value; rather, they indicate opportunities to extend their reach and effectiveness beyond traditional regulatory environments.

## Beyond the ear: Social determinants and multi-hazard exposures

Hearing loss is deeply embedded within the social determinants of health. Poverty limits access to protective equipment, hearing healthcare services and hearing assistive technologies (Jilla et al., 2023), while lower education levels and limited occupational hearing health awareness may reduce recognition of hazardous noise exposure, appropriate use of hearing protection and workplace advocacy (Tikka et al., 2017). Geographic disparities, particularly in rural and underserved communities, further exacerbate delays in detection and intervention. In addition, stigma associated with hearing loss and hearing aid use may suppress help-seeking behaviours even where services are available (Knoetze et al., 2023).

In Southern Africa, the convergence of occupational noise with HIV and tuberculosis further amplifies vulnerability (Fokouo et al., 2015; Khoza-Shangase, 2025b). Ototoxic therapies and opportunistic infections may accelerate auditory damage, creating dual-burden vulnerabilities that require integration of routine audiological monitoring within occupational and HIV care systems (Khoza-Shangase, 2025b). These intersecting exposures highlight the limitations of conventional HCPs that focus primarily on noise measurement while insufficiently accounting for broader occupational, biological and social risk profiles. Addressing ONIHL in SSA therefore requires structural interventions beyond regulatory compliance alone. These include strengthening universal health coverage (UHC), workplace health literacy initiatives, community-linked prevention strategies, destigmatisation campaigns and inclusive hearing device design.

## From compliance to innovation: Embedding equity through Fourth Industrial Revolution technologies

Reframing ONIHL prevention around innovation and equity requires technology to function as a tool for democratising expertise, improving continuity of care and strengthening existing HCPs rather than replacing them. Fourth Industrial Revolution technologies offer several potentially transformative pathways:

- **Tele-audiology and mHealth:** Remote assessments and smartphone-based screening platforms can extend services to rural, informal and underserved populations while reducing geographic and infrastructural barriers (Clark & Swanepoel, 2014; D’Onofrio & Zeng, 2022). South African initiatives such as hearX Group demonstrate scalable and comparatively low-cost decentralised hearing testing approaches (Frisby et al., 2022). These approaches may strengthen existing HCPs by improving access to monitoring and follow-up services.
- **AI and predictive analytics:** AI-enabled systems may support predictive risk surveillance by identifying high-risk individuals and occupational environments before permanent hearing damage occurs. Integrating occupational, environmental and clinical data can facilitate earlier intervention and strengthen continuity of prevention efforts beyond annual compliance testing – thus supporting anticipatory, rather than reactive intervention (Madahana et al., 2024).
- **IoT and connected devices:** IoT-enabled dosimeters, smart hearing protection devices and environmental sensors allow real-time exposure monitoring and hazard feedback (Chis et al., 2025; Madahana et al., 2024; Moroe et al., 2019). Cloud-based analytics may support more dynamic risk management approaches and strengthen regulatory monitoring systems (Madahana et al., 2024).
- **Big data and compensation analytics:** Analysis of occupational compensation datasets (e.g. Rand Mutual Assurance) may assist in identifying high-risk sectors, informing revisions to exposure limits, strengthening surveillance systems and supporting targeted occupational interventions (Heberger & Wurzelbacher, 2024).
- **Community health worker (CHW) integration:** Embedding CHWs within tele-audiology and community-linked prevention initiatives may enhance culturally and linguistically responsive counselling, strengthen follow-up and improve digital inclusion (Coco et al., 2021). Such approaches may help bridge gaps between formal occupational systems and underserved communities. These approaches are also consistent with the WHO World Report on Hearing, which advocates integrated, community-oriented hearing care and strengthened primary healthcare platforms as mechanisms for expanding equitable access (WHO, 2021).

Collectively, these approaches support a more distributed, data-informed and equity-centred hearing conservation ecosystem extending beyond formal workplaces and into broader community contexts. Crucially, implementation of 4IR technologies within SSA must account for infrastructural and contextual limitations. In many rural and underserved settings, Internet connectivity, electricity supply, device availability and digital literacy remain inconsistent (Agbeyangi & Lukose, 2025). Consequently, fully digital models may not be feasible across all contexts. Hybrid implementation approaches that combine digital technologies with workplace-based services, offline-compatible tools and community-supported models may therefore provide more contextually responsive pathways for strengthening ONIHL prevention.

## Regulatory reform, trade unions and enforcement

Technological innovation alone is unlikely to improve prevention outcomes without parallel regulatory and systems-level reform. Rather than replacing existing occupational HCPs, the proposed approach seeks to strengthen and modernise current systems through digital augmentation, improved surveillance and enhanced continuity of care. Existing occupational noise regulations and HCP frameworks have improved surveillance and awareness in some industries; however, implementation and enforcement remain inconsistent across jurisdictions and sectors (Ibili et al., 2023; Khoza-Shangase, 2025e, 2025f). Exposure limits, monitoring systems and compensation frameworks continue to vary substantially across African settings, often prioritising documentation and compensation processes over proactive prevention and continuity of care. Furthermore, many occupational monitoring systems remain reactive, relying predominantly on periodic testing rather than continuous or predictive surveillance approaches. Trade unions may represent an important but underutilised mechanism for strengthening hearing health advocacy, promoting use of personal protective equipment, supporting worker education and advocating for engineering controls within hazardous environments (Khoza-Shangase, 2025f; Muthelo et al., 2022). Extending prevention to underserved occupational groups will also require pragmatic surveillance approaches that generate reliable exposure and hearing health data to inform policy, programme evaluation and resource allocation (WHO, 2021). Strengthening collaboration between occupational health practitioners, audiologists, labour structures, employers and policymakers may therefore support more accountable and prevention-oriented HCP implementation. Moving from paper-based compliance systems towards active, technology-supported enforcement and integrated surveillance may strengthen both prevention and accountability across occupational settings. Future implementation should also incorporate measurable indicators such as hearing protection utilisation, early threshold shift detection, programme coverage and worker-centred outcome measures to evaluate effectiveness.

## Governance, digital sovereignty and ethical leadership

The deployment of 4IR technologies raises important governance and ethical considerations. Data privacy, algorithmic fairness and equitable access should be prioritised to reduce the risk of reproducing existing inequalities through digital systems (Khoza-Shangase, 2025d). For this reason, ethical governance frameworks are necessary to support responsible implementation of AI-driven and data-intensive hearing healthcare systems. African nations must pursue digital sovereignty by strengthening local control over health data systems, digital infrastructure and AI development processes (Dos Santos et al., 2025). Reliance on datasets developed predominantly within Global North populations may increase the risk of diagnostic inaccuracies and cultural or linguistic bias in African settings (Grancia, 2025). Investment in locally generated datasets and African-led innovation ecosystems is therefore important for contextually appropriate implementation. Policy harmonisation, ethical oversight and intersectoral leadership are needed to embed ONIHL prevention within broader national digital health strategies, Sustainable Development Goals (SDGs) and African Union frameworks. Within this process, audiologists should be positioned not only as clinicians but also as contributors to prevention policy, occupational health innovation and equitable digital health implementation.

## Conclusion: Towards an innovation-led, equity-driven future

The persistence of ONIHL in SSA reflects limitations within predominantly compliance-focused frameworks. Preventing this avoidable occupational condition requires a broader reorientation towards innovation, health equity and contextually responsive technological integration. Existing HCPs and occupational regulations have contributed to surveillance and awareness within some sectors, particularly mining. However, important gaps remain in predictive risk monitoring, multi-hazard integration, continuity of care and inclusion of informal and underserved worker populations. Fourth Industrial Revolution technologies provide opportunities to strengthen existing systems through decentralised service delivery, real-time exposure monitoring, predictive analytics and community-linked prevention approaches. At the same time, implementation must remain responsive to infrastructural realities, digital inequities and ethical governance considerations within SSA. Strengthening ONIHL prevention will require phased implementation that combines strengthened policy implementation, contextually appropriate 4IR technologies, community-based service delivery and routine evaluation of prevention outcomes. Reframing ONIHL prevention as an equity-driven and innovation-oriented public health priority positions SSA not merely as a consumer of imported models but as a contributor to contextually responsive, sustainable and socially just hearing conservation approaches. Protecting hearing health is therefore not solely a regulatory obligation but also a commitment to dignity, productivity and equitable occupational health futures for noise-exposed workers. As an opinion paper, this article is intended to stimulate discussion rather than provide a systematic review of all occupational hearing conservation policies or evaluate the effectiveness of individual programmes. Future empirical research should examine implementation strategies, policy effectiveness and measurable outcomes associated with integrating 4IR technologies into occupational hearing conservation across diverse African contexts.
